# Patellofemoral cartilage defects are acceptable in patients undergoing high tibial osteotomy for medial osteoarthritis of the knee

**DOI:** 10.1186/s12891-022-05398-3

**Published:** 2022-05-24

**Authors:** Lisa Bode, Jan Kühle, Anna-Sophie Brenner, Viola Freigang, Helge Eberbach, Philipp Niemeyer, Norbert P. Südkamp, Hagen Schmal, Gerrit Bode

**Affiliations:** 1 Department of Orthopedics and Trauma Surgery, Faculty of Medicine, Medical Center - Albert-Ludwigs-University of Freiburg, Albert-Ludwigs-University of Freiburg, Freiburg, Germany; 2 Department of Trauma Surgery, University Hospital Regensburg, Franz-Josef-Strauss-Allee 11, 93053 Regensburg, Germany; 3 OCM Clinic, Munich, Germany; 4 Department of Orthopedic Surgery, University Hospital Odense, Sdr. Boulevard 29, 5000 Odense C, Denmark; 5 Praxisklinik 2000, Wirthstr. 11A, Freiburg, Germany

**Keywords:** Knee, High tibial osteotomy, Varus deformity, Patella, Cartilage defects

## Abstract

**Background:**

Patients suffering cartilage defects of the medial compartment with underlying varus deformity do benefit from high tibial osteotomy (HTO) even in the long term. Nonetheless, kinematic and geometric changes especially in the patellofemoral joint have been described.

Purpose of the present study was to evaluate the influence of patellofemoral cartilage defects detected during the diagnostic arthroscopy and their influence on HTO’s postoperative outcome.

**Methods:**

Ninety patients with a mean follow-up of 10.08 ± 2.33 years after surgery were included. Patients were divided into four groups according to their cartilage status in the patellofemoral joint (A = no defects, B = isolated lesions of the patella, C = isolated lesions of the trochlea, D = kissing lesions). Functional outcome was evaluated before surgery and about ten years thereafter by relying on the IKDC, Lysholm, and KOOS scores. Radiological parameters were assessed pre- and six weeks postoperatively.

**Results:**

In groups A to D, the HTO led to significant patellar distalisation in the sagittal view, with the mean indices remaining at or above the limit to a patella baja. All patients in all groups profited significantly from HTO (higher Lysholm score, lower VAS *p* < 0.001), patients in group D had the lowest outcome scores. Patella height negatively influenced outcome scores in group C (Blackburne-Peel-Index—VAS *p* = 0.033) and D (Caton-Deschamps-Index—Tegner *p* = 0.018), a larger valgus correction was associated with lower outcome scores in group D (Lysholm *p* = 0.044, KOOSpain 0.028, KOOSQOL *p* = 0.004).

**Conclusion:**

Long-term results of HTO for varus medial compartment osteoarthritis remain good to excellent even in the presence of patellofemoral defects. Overcorrection should be avoided. Distal biplanar HTO should be considered for patients presenting trochlear or kissing lesions of the patellofemoral joint.

**Trial registration:**

DRKS00015733 in the German Registry of Clinical Studies.

## Background

Medial high tibial open-wedge osteotomy (OW-HTO) has proven to be a safe and efficient surgical therapy with good to excellent results for patients suffering degenerative impairments in the medial compartment [1].

Even long-term results are promising [2]. Nonetheless, OW-HTO remains a demanding intervention with notable risk factors [3]. While risk factors were significantly reduced applying standard surgical techniques [4–6] patellofemoral joint anomalies remain a serious postoperative problem [7, 8].

A loss of patellar height followed by degeneration has been highlighted, but the latest published evidence is inconclusive. While several studies report worsening preoperative patellar defects following HTO, no significant influence on the postoperative functional outcome has also been observed [9, 10].

While there is broad consensus on performing biplanar HTO in the tibia’s distal tuberosity in conjunction with high-grade cartilage defects of the patella [11, 12] there is very little data on the impact of the low-grade cartilage defects often detected during standard arthroscopy prior to HTO. Degenerative patellofemoral cartilage defects used to be a contraindication for HTO [13]. Unfortunately, these patients also carry a high risk for complication rates following total knee arthroplasty (TKA) [14]. Modified surgical techniques such as a distally adverted OW-HTO can benefit patients suffering from medial and patellofemoral OA, especially when they face a therapeutic gap (e.g. young and active patients aiming at avoiding knee arthroplasty) [15].

Modified techniques tending to lower patellofemoral joint (PFJ) pressure have been described and their advantages proven in biomechanical tests [16, 17]. Such techniques are recommended for patients with medial compartment osteoarthritis and patellofemoral pain syndrome, but there are few recommendations for early-stage cartilage defects or mild degenerative anomalies. Jungmann et al. recently pointed out that untreated cartilage defects both tend to progress and exacerbate the degeneration throughout the joint [18].

Therefore, even early-stage cartilage defects of the patella must be considered when planning OW-HTO in order to unload the medial compartment without loading the anterior compartment.

In this study, we hypothesised that patellofemoral joint cartilage defects are associated with worse patient reported outcomes (PROMs) when performing biplanar ascending OW-HTO. Second hypothesis was that the patellofemoral joint’s postoperative radiological parameters (Caton-Deschamps Index, Blackburne-Peel Index, Insall-Salvation-Index) remain within physiological ranges if a mild correction of 2–3° valgus is chosen which might lead to better PROMs than in cases of a larger valgus correction.

## Method

**Fig. 1:**
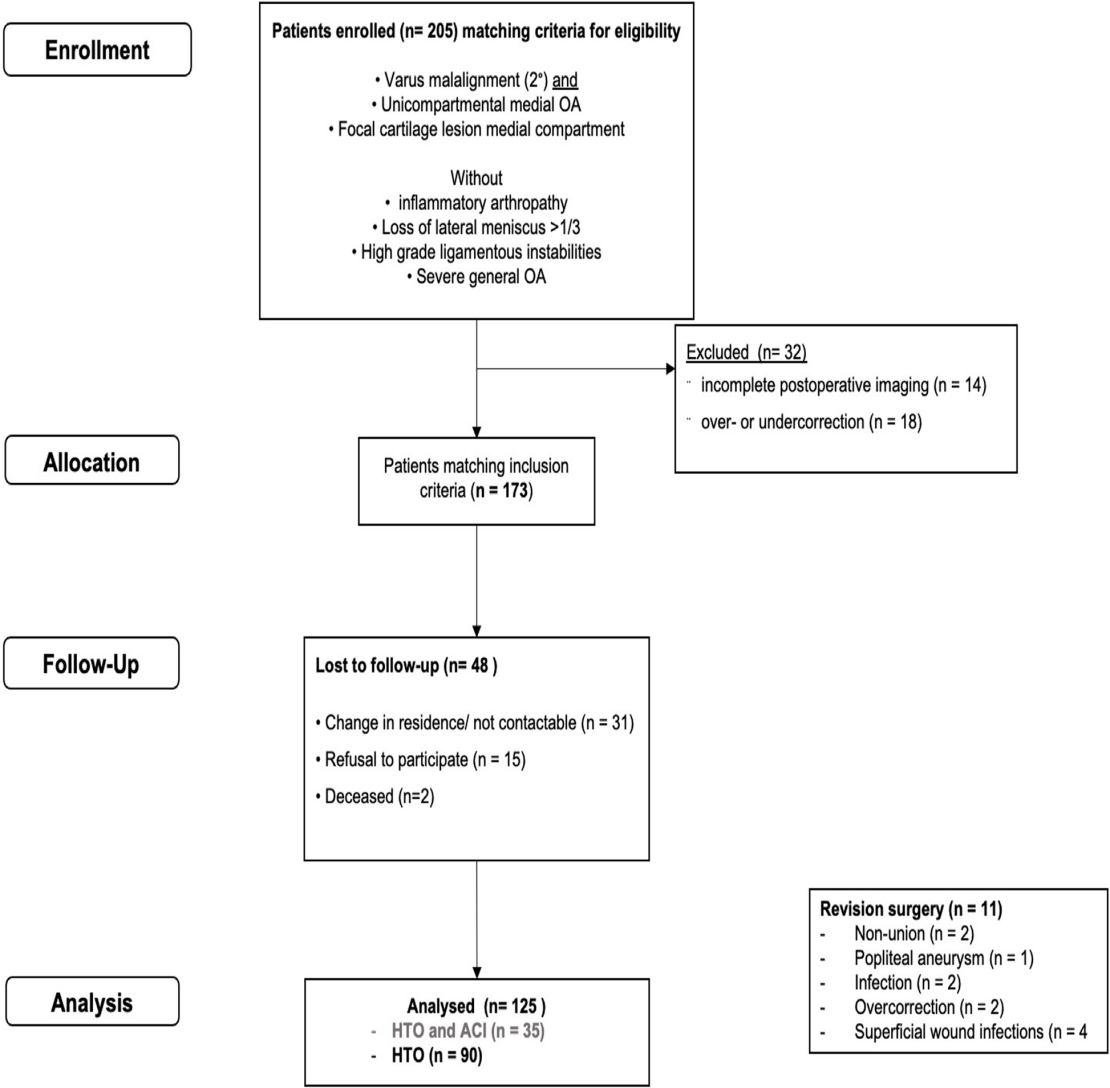
Patient Flow Chart. Data on patients excluded from the study who had refused to participate, died, or were not reachable. Patients with HTO and ACI were excluded for this analysis

Study design of the present study aimed to examine the influence of patellofemoral cartilage defects on biplanar ascending OW-HTO’s long-term functional outcome using angular stable internal plate fixation for medial osteoarthritis with concomitant varus deformity. Clinical outcome of the cohort was registered prospectively and the cohort treated by OW-HTO for medial OA was additionally analyzed during the 10-year’s follow-up survey [2] for potential effects of concomitant patellofemoral cartilage lesions on the clinical and functional outcome in this study. Therefore, ninety patients were enrolled in an orthopaedic and trauma surgery clinic from 1/1/2004 to 31/12/2013 who underwent OW-HTO using an angular stable internal plate fixator (Tomofix Synthes Switzerland) for medial osteoarthritis with concomitant varus deformity for follow-up examinations (Flow Chart, Fig. 1), as they were eligible for this study and met the inclusion criteria as described in previous studies [2, 19–21]:

Before their OW-HTO, patients underwent one-stage arthroscopy to assess the medial, lateral, and patellofemoral compartments; patients with severe degenerative anomalies were excluded. Patients were ineligible for the study if there was any significantly restricted flexion, as well as if they suffered from inflammatory arthropathy, extensive loss (> 2/3 of its surface) or absence of the lateral meniscus or high-grade ligamentous instabilities, or from severe general osteoarthritis (OA) including the lateral and patellofemoral compartment. Patients were asked before surgery about their pain in the lateral compartment. If they felt pain, they did not qualify for medial open-wedge osteotomy. Smoking was not a contraindication except in case of severe nicotine abuse (over 20 pack years). Patients aged up to 70 years were included at the time of surgery. Their range of motion had to be at least a flexion/extension of 120–0° [2]. Extension deficits were integrated in preoperative planning.

### In- and exclusion criteria

Patients presenting focal cartilage lesions of the lateral or patellofemoral compartment were included in the study if they fulfilled enrolment criteria.

Exclusion criteria were: postoperative over- or undercorrection of the leg axis (defined as a correction exceeding the 65%- or undercutting of the 50%- intersection point on the tibial plateau, if the medial border of the tibial plateau represents the 0%-intersection point and the lateral border the 100%-intersection point of the weight bearing line six weeks postoperatively), incomplete or unavailable postoperative imaging, failure to provide informed consent, or not available for follow-up evaluation (Fig. 1). For the current analysis of patellofemoral cartilage lesions, patients who underwent autologous chondrocyte implantation (ACI) and HTO for focal medial compartment cartilage damage were excluded; only those who received HTO for medial OA were included (Fig. 1).

### Preoperative management and surgical technique

The necessary preoperative diagnostics have been described [19–21] and are restated here: limb alignment was assessed via the Paley technique [22], including measuring the mechanical lateral distal femoral angle (mLDFA), mechanical medial proximal tibial angle (mMPTA), and joint line convergence angle (JLCA).

Patients were given general anaesthesia, intravenous antibiotics, and standard thromboembolic prophylaxis. After routine arthroscopy, HTO was performed according to the technique recommended by the international knee expert group as described [3, 6]. The extent of preoperatively planned correction was intraoperatively controlled via a navigation system (Orthopilot™; Aesculap Co. Tuttlingen, Germany; Software: Orthopilot software for HTO). All osteotomies were done in biplanar manner and stabilised using the Tomofix™ system (Tomofix™, Solothurn, Synthes) and correction aimed to achieve a mild valgus alignment [19]. Postoperative mobilisation started on day one, and continuous passive motion was recommended for the first six weeks lasting 4–6 h daily. Limited weight-bearing was allowed three weeks postoperatively. Patients were not limited in their range of motion at any time. Once full weight-bearing was achieved, full-leg radiographs were taken to analyze the postoperative weight-bearing axis. Digital analyses of pre- and postoperative full-leg radiographs were done before the statistical analyses (mediCAD, Hectec GmbH, Germany).

Patient characteristics of the entire cohort are displayed in Table 1.

**Table 1:** Patient characteristics of the entire cohort (*n* = 90). Preoperative radiological values: varus deformity, Caton-Deschamps-Index (CDI), Blackburne-Peel-Index (BPI) and Insall-Salvati-Index (ISI)

n	90
**Age at surgery (years, mean, SD, range)**	46.64 ± 9.87 (18,90 – 66.90)
**Sex (f: m)**	31: 59
**Follow up (months, mean, SD, range)**	121.0 ± 28.03 (73 – 171)
**BMI at surgery (kg/ m**^**2**^**)**	27.75 ± 4.64 (12.30 – 41.30)
**Symptoms prior to surgery (months)**	26.68 ± 36.27 (2 – 240), median 15.0
**Varus preOP (°)**	6.37 ± 2.76 (0.90 – 14.10)
**CDI preOP**	1.04 ± 0.16 (0.66 – 1.5)
**BPI preOP**	0.94 ± 0.17 (0.31—1.3)
**ISI preOP**	0.92 ± 0.15 (0.70 – 1.40)

Patients were then divided into four subgroups according to their patellofemoral cartilage lesions. While group A presented no cartilage lesions in the one-stage arthroscopy before HTO, patellar and trochlear lesions were detected in groups B and C, respectively. Group D consisted of patients with kissing cartilage lesions in the patellofemoral joint.

### Clinical outcome, radiological outcome parameters and survival

Patient interviews took place between February and July 2019 after they had provided written consent for study participation. Follow-up was defined as the time period from the day of surgery until the day of interview. Functional outcome was evaluated by applying the standard Lysholm Score (pre- and postoperatively), International Knee Documentation Committee score (IKDC), Tegner Score (pre- and postoperative), and the Knee and Osteoarthritis Outcome Score (KOOS), as well as the Western Ontario and McMaster Universities Osteoarthritis Score (WOMAC Score). Pre- and postoperative pain levels were evaluated by the Visual Analogue Scale (VAS) [23–25]. The authors conducted the interview by phone. Further outcome parameters in this study included the comparison and acquisition of radiological parameters in the sagittal and frontal plane (detailed results of the analysis in the frontal plane have been published) [2]. Preoperative varus deformity and postoperative valgus correction were defined as medial or lateral deviation from the mechanical weight-bearing axis (hip-to-ankle line through the center of the knee). A retrospective analysis of the anteroposterior weight-bearing long-leg view was done by documenting the intersection point of the weight-bearing line with the tibial plateau (TP). The tibial plateau’s medial border represented 0% and the lateral border 100%. Target for all patients was to hit the TP between 50 – 65%.

Later the patella height was measured in the sagittal view and was compared using the Insall-Salvati (ISI)-, Blackburne-Peel- (BPI), and Caton-Deschamp indices (CDI) as described above. Complications were recorded (Fig. 1) and classified as any major or minor complication leading to revision surgery. Major complications included popliteal aneurysm, large overcorrection resulting in immediate revision, delayed union and deep-tissue infections; delayed wound healing was defined as a minor complication. Any discomfort caused by the implant was also recorded.

### Statistical analysis

SPSS for Windows (Version 27; SPSS, Chicago IL) was used for statistical analysis. Quantitative variables at baseline were expressed as mean ± standard deviation (SD). An explorative analysis of the subgroups was performed. Normal distribution was assessed with the Kolmogorov–Smirnov test. Categorical variables were compared by the chi-square test. For multiple testing ANOVA (normally distributed data) or Kruskal–Wallis test (non-normally distributed data) were used. In case of multiple testing the Bonferroni correction was added. For comparison of normally distributed paired samples students’ t-test was applied. Fixed effects logistic regression was used to estimate associations between the dependent variable patellofemoral lesion (yes/no) and the following independent variables: patients’ characteristics (age, BMI at the time of surgery, sex), postoperative radiological parameters (valgus angle, CDI, BPI) and functional scores (KOOS subscores, IKDC, VAS, Lysholm Score, KOOS subscores, WOMAC subscores, Tegner Score). Pearson’s (for normally distributed data) and Spearman’s correlation (for not-normally distributed data) was used to measure associations between two variables. Accordingly, *p* < 0.05 was considered statistically significant.

#### Ethics approval

The ethics committee of Freiburg University approved this study (ID 290/18).

## Results

**Fig. 2:**
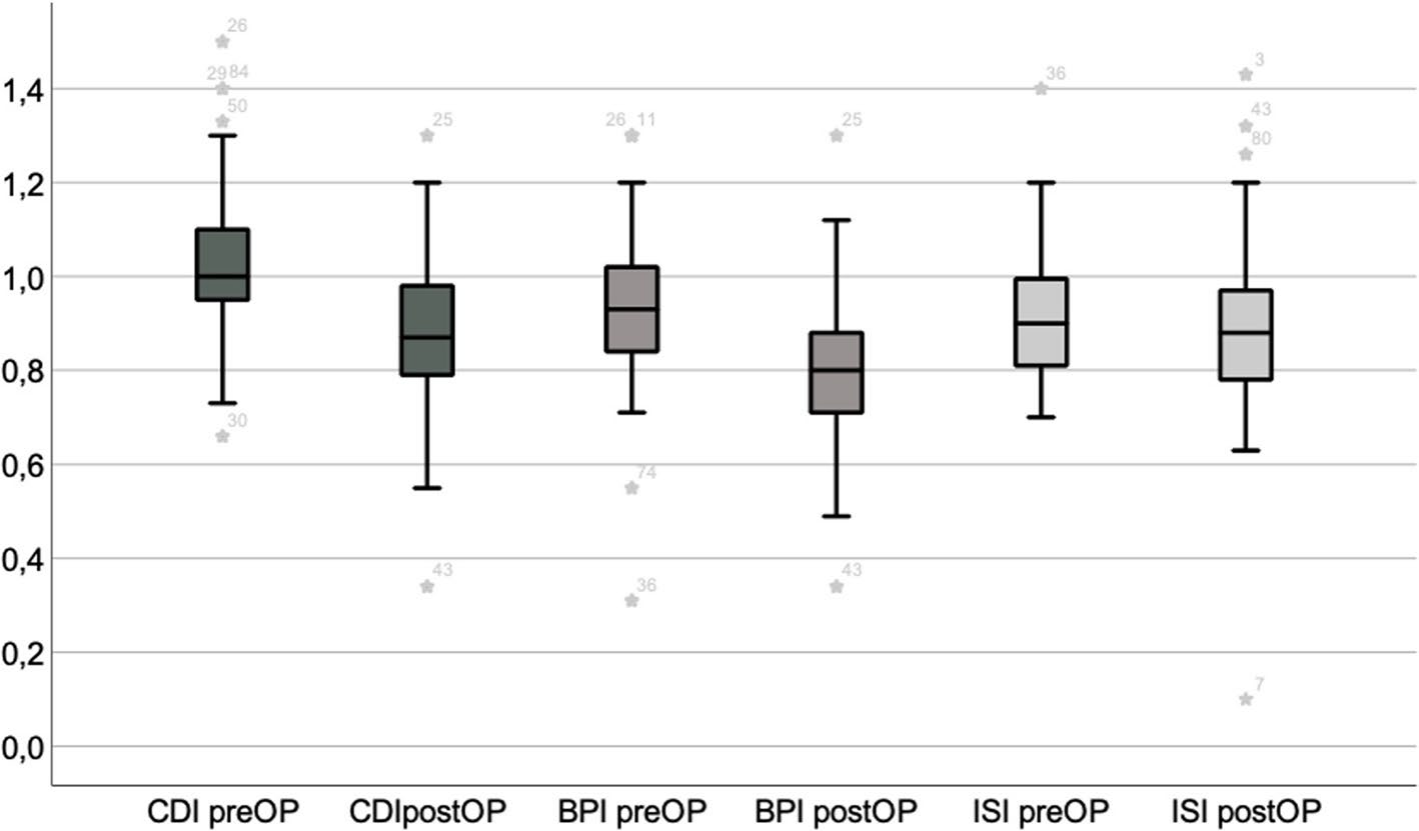
Pre- to postoperative CDI and BPI values changed significantly with a postoperative CDI of 0.88 ± 0.17 (95% CI 0.85 – 0.92)* and postoperative BPI of 0.80 ± 0.16 (95% CI 0.77 – 0.84)*. The ISI did not change significantly

Statistical analysis of the entire cohort revealed a postoperative valgus of 2.69° ± 1.73 (95% Confidence Intervall, CI, 2.32 – 3.06°). Indices of patella height changed significantly after HTO but remained within the physiological range (Fig. 2). Lysholm (43.47 ± 20.09, 95% CI 39.26 – 47.68 to 80.17 ± 17.67, 95% CI 76.47 – 83.87), VAS (7.33 ± 1.68, 95% CI 6.98 – 7.69 to 2.96 ± 2.55, 95% CI 2.42—3.49) and Tegner Scores (5.26 ± 1.20, 95% CI 5.0 – 5.51 to 3.68 ± 1.64, 95% CI 3.33–4.02) changed significantly preoperatively to postoperatively (p < 0.001). Additional postoperative data on the cohort are displayed in Table 2.

**Table 2:** Data on absence from work after HTO and implant removal. Median was added as the time span varied broadly. The cohort’s functional scores showed good to excellent results concerning symptoms, function, pain, daily living and quality of life, and moderate results concerning the ability to engage in sports at the final follow-up

*N* = 90	Mean values ± SD (range)
**Absence from work after HTO (days)**	89.54 ± 82.72 (6 – 540), median 70.0
**Absence from work after implant removal (days)**	19.65 ± 20.08 (4 – 90), median 14.0
**IKDC 10y**	64.11 ± 19.39 (23 -100)
**KOOSpain 10y**	81.97 ± 15.36 (47.20 – 100)
**KOOSsymp 10y**	77.10 ± 18.39 (17.90 – 100)
**KOOSadl 10y**	86.38 ± 14.22 (36.80 – 100)
**KOOSsport 10y**	51.17 ± 30.83 (0 – 100)
**KOOSQOL 10y**	70.26 ± 22.42 (6.30 – 100)
**KOOS4 10y**	70.13 ± 19.22 (22.50 – 100)
**WOMACpain 10y**	2.72 ± 3.02 (0 – 12)
**WOMACstiff 10y**	1.22 ± 1.75 (0 – 7)
**WOMACfunction 10y**	9.12 ± 9.56 (0 – 43)

### Subgroup analysis

The subgroups differed significantly in age at the time of surgery (A vs.D *p* = 0.014) and pre-operative BPI (Group C vs. A *p* = 0.026, group C vs. D *p* = 0.020). Patients’ characteristics in all four subgroups are displayed in Table 3.

**Table 3:** Patient characteristics in all four subgroups (A no patellofemoral defects, B patella surface defects, C trochlear defects, D kissing lesions in the patellofemoral joint). Pre- and six weeks postoperative radiological values: varus deformity and valgus correction, as well as preop CDP, BPI and ISI

Group	A	B	C	D
**n**	39	20	9	22
**Age at surgery (years, mean, SD, range)**	42.72 ± 10.76* (18.90 – 57.20)	49.34 ± 8.05 (33.20 – 62.20)	47.11 ± 6.68 (38.30 – 56.90)	50.93 ± 8.56 (27.60 – 66.90)
**Follow-up (years, mean, SD, range)**	119.87 ± 29.32 (73 – 171)	130.5 ± 27.15 (86 – 171)	123 ± 30.94 (77 – 166)	113.55 ± 24.38 (78 –167)
**BMI at surgery (kg/m**^**2**^**)**	26.34 ± 4.64 (12.30 – 37.90)	28.78 ± 4.15 (20.80 – 36.80)	27.76 ± 3.45 (23.30 – 35.20)	29.32 ± 5.01 (20.70 – 41.30)
**Varus preOP (°)**	6.12 ± 2.86 (2.1 – 13.30)	7.33 ± 2.80 (4.2 – 14.10)	6.76 ± 1.72 (4.50 – 9.30)	5.82 ± 2.83 (2 – 13.90)
**Valgus postOP (°)**	3.16 ± 1.77 (0.3 – 10.60)	2.20 ± 1.59 (0.1 – 5.6)	2.76 ± 1.83 (0.9 – 5.8)	2.31 ± 1.67 (0.1 – 7.2)
**CDI preOP**	1.02 ± 0.18 (0.73 – 1.5)	1.06—± 0.13 (0.8 – 1.3)	1.17 ± 0.18 (0.95 – 1.4)	0.99 ± 0.12 (0.66 – 1.2)
**BPI preOP**	0.91 ± 0.22 (0.31- 1.3)	0.95 ± 1.11 (0.78 – 1.1)	1.08 ± 0.95 (0.94 – 1.2)*	0.91 ± 0.13 (0.75 – 1.3)
**ISI preOP**	0.92 ± 0.15 (0.71 – 1.40)	0.87 ± 0.14 (0.7 – 1.1)	0.95 ± 0.15 (0.76 – 1.2)	0.95 ± 0.15 (0.71 – 1.2)

Significant results are marked by *

**Fig. 3:**
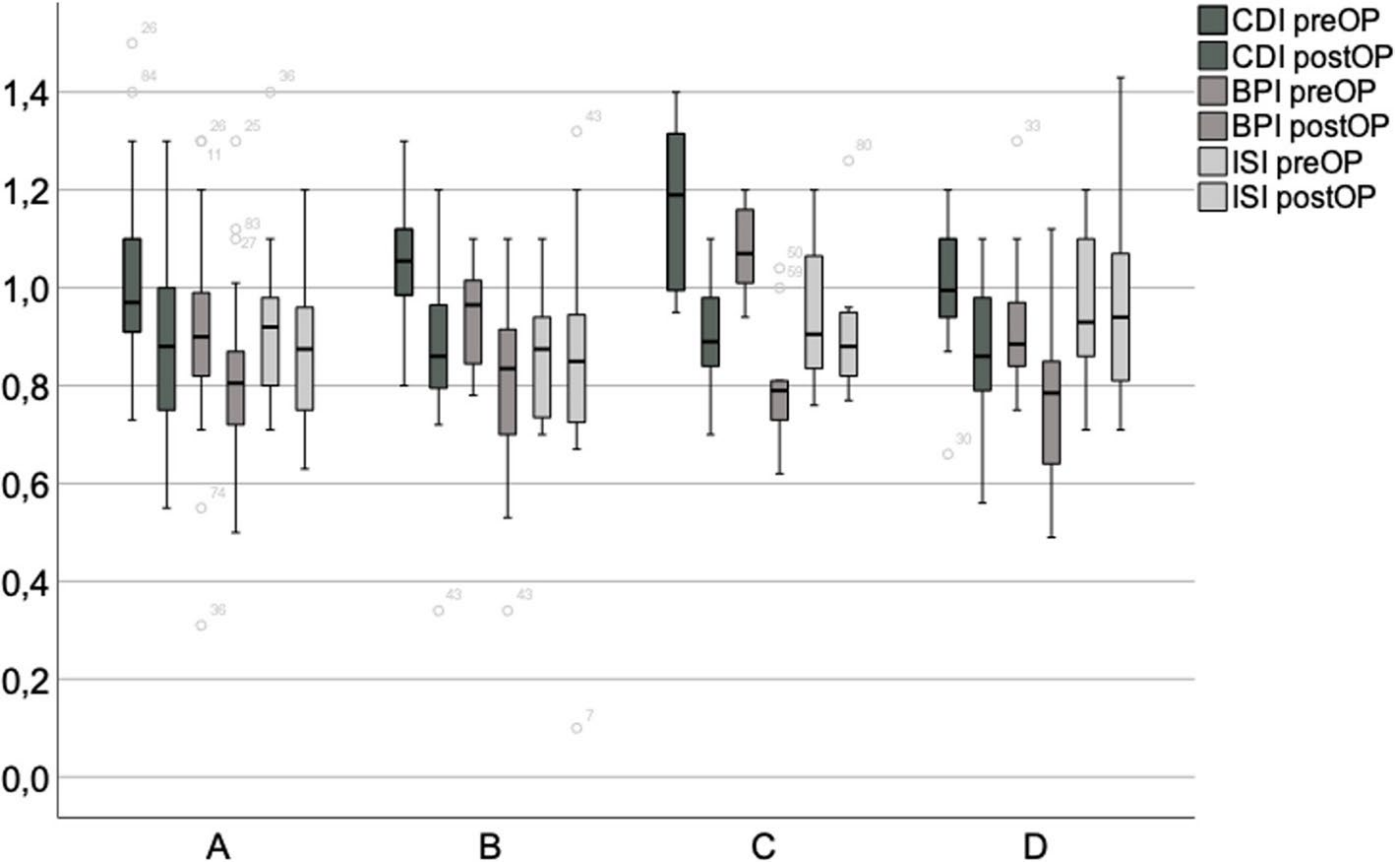
Patella distalisation in groups A – D. Significant changes from pre- to postoperative values were observed in group A (CDI *p* = 0.006), B (CDI *p* = 0.001, BPI *p* = 0.006), C (CDI *p* < 0.001, BPI *p* < 0.001) and D (CDI *p* < 0.001, BPI *p* = 0.001)

Similar to the entire cohort, HTO led to the patella’s significant distalisation in the subgroups as well, with the mean indices remaining at or above the limit to a patella baja (Group A mean CDI 0.90 ± 0.17, BPI 0.82 ± 0.15, Group B CDI 0.89 ± 0.21, BPI 0.80 ± 0.19, Group C CDI 0.90 ± 0.12, BPI 0.81 ± 0.13, Group D CDI 0.85 ± 0.14, BPI 0.78 ± 0.15), cf. Figure 3. There were no significant differences between Groups A and D in postoperative patellar height.

**Fig. 4:**
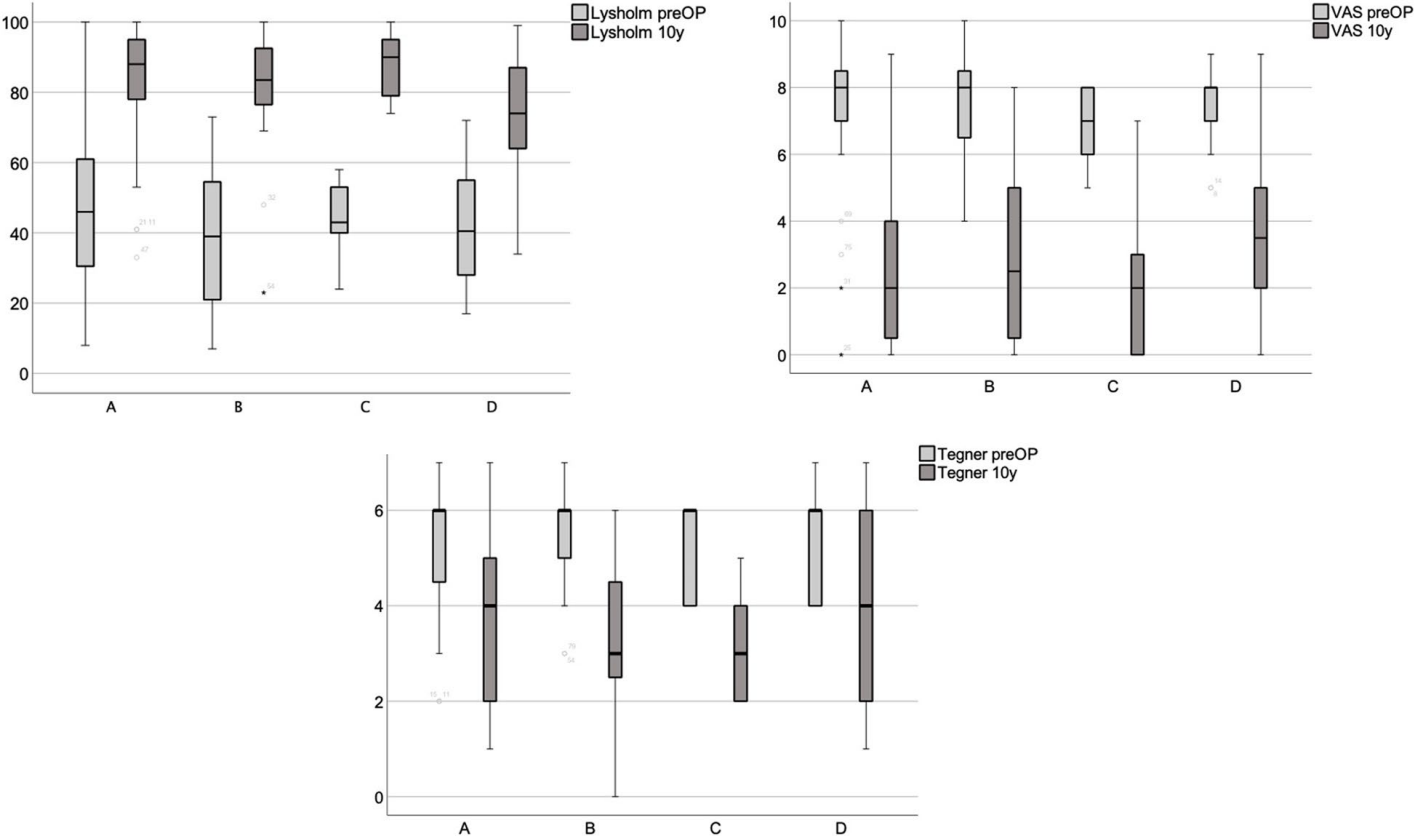
Groups A to D patients rise in Lysholm Scores and lower VAS pain levels. Sports and work activity levels fell significantly (Tegner pre- to postOP Groups A – D *p* < 0.001)

Patients in all groups showed pre- to postoperatively a significant rise in the Lysholm Score (Groups A to D *p* < 0.001), and lower pain levels (VAS Score, groups A to D *p* < 0.001). Group D patients’ Lysholm Score was significantly lower at final follow-up than that of patients in group A (*p* = 0.038). Tegner Scale assessments of activity levels in sports and work revealed lower levels (Fig. 4).

Regarding the IKDC, KOOS and subscores (cf. Table 4): group D patients achieved significantly lower KOOS quality of life score (*p* = 0.023) than group A. Absolute WOMAC subscores were low in all four groups, indicating good functional results in all concerning pain, stiffness, and function and did not significantly differ between the groups.

**Table 4:** Functional scores in all four subgroups

	**A**	**B**	**C**	**D**
**n**	39	20	9	22
**BMI 10y(kg/m**^**2**^**)**	26.73 ± 3.93 (20.40 – 38.30)	29.08 ± 4.25 (21.80 – 35.30)	28.76 ± 4.29 (21.60 – 37.0)	28.67 ± 4.56 (20.20 – 37.50)
**Outerbridge Score femoropatellar cartilage lesion**	-			Patellar/ trochlear
		1: *n* = 0	1: *n* = 2	1: *n* = 2 / *n* = 1
		2: *n* = 10	2: *n* = 3	2: *n* = 8/ *n* = 8
		3: n = 9	3: *n* = 2	3: *n* = 9/ *n* = 10
		4: *n* = 1	4: *n* = 2	4: *n* = 3/ *n* = 3
**IKDC 10y**	67.65 ± 20.62 (23 – 100)	63.0 ± 19.03 (26.4 – 92.0)	66.03 ± 11.9 (44.8 – 82.8)	58.06 ± 19.44 (24.1 – 88.5)
**KOOSpain 10y**	83.83 ± 16.10 (47.2 – 100)	81.66 ± 13.35 (47.2 – 100)	88.89 ± 10.65 (66.7 -100)	76.14 ± 16.26 (47.2 – 100)
**KOOSsymp 10y**	79.03 ± 19.28 (32.1- 100)	82.87 ± 12.67 (60.7 – 100)	75.01 ± 13.49 (60.70 -100)	69.32 ± 21.03 (17.90 – 96.40)
**KOOSadl 10y**	88.80 ± 13.64 (50 – 100)	86.47 ± 14.98 (36.80 – 100)	88.42 ± 7.65 (80.9 – 100)	81.15 ± 15.93 (51.5 – 100)
**KOOSsport 10y**	58.08 ± 33.28 (0 – 100)	46.75 ± 29.57 (0 – 100)	48.89 ± 21.47 (15 – 80)	43.86 ± 29.88 (0 – 100)
**KOOSQOL 10y**	75.34 ± 21.16 (18.8 – 100)	71.59 ± 20.12 (18.8 – 100)	77.81 ± 11.73 (56.30 – 93.80)	56.97 ± 25.34* (6.3 – 93.8)
**KOOS4 10y**	74.07 ± 20.54 (29.2 – 100)	70.72 ± 16.03 (33.8 – 96.6)	72.66 ± 10.90 (65.10 – 91.88)	61.58 ± 20.38 (22.5 – 95.98)
**WOMACpain**	2.38 ± 3.08 (0 – 10)	2.95 ± 2.95 (0—12)	1.11 ± 1.54 (0 – 4)	3.77 ± 3.21 (0 – 11)
**WOMACstiffness**	1.21 ± 1.73 (0 – 5)	0.7 ± 1.17 (0 – 3)	0.44 ± 0.88 (0 – 2)	2.05 ± 2.19 (0 – 7)
**WOMACfunction**	7.14 ± 9.14 (0 – 34)	9.15 ± 10.17 (0 – 43)	7.89 ± 5.21 (0 – 13)	12.64 ± 10.63 (0 – 33)

Significant results are marked by *

### Correlations in subgroups with patellofemoral cartilage lesions

Group B. In this subgroup, a correlation between the retropatellar cartilage lesion’s Outerbridge classification and KOOSsymp (*p* = 0.006, *r* = -0.595**) and WOMACstiffness scores (*p* = 0.022, *r* = 0.508) could be observed. Female sex predisposed for a lower KOOSpain score (*p* = 0.026, *r* = -0.495) and higher WOMACpain (*p* = 0.011, *r* = 0.557) and WOMACstiffness scores (*p* = 0.014, *r* = 0.528). A higher BMI at the time of surgery was a risk factor for a longer absence from work after HTO (*p* = 0.008, *r* = 0.607**). No correlation between postoperative CDI, BPI, and ISI could be detected.

Group C. A larger postoperative valgus was associated with a lower CDI (*p* = 0.01, *r* = -0.833). A lower BPI led to higher VAS pain levels at final follow-up (*p* = 0.033, *r* = -0.707). The higher the difference between pre- and postoperative ISI, the lower the Lysholm Score at final follow-up (*p* = 0.047, *r *= -0.714).

Group D. A higher postoperative CDI was associated with a higher Tegner Score (*p* = 0.018, *r* = 0.499). A milder valgus correction measured in % on the tibia plateau (TP) in this group was associated with superior functional results (correction TP%—Lysholm Score *p* = 0.044, *r* = -0.433, correction TP%—KOOSpain *p* = 0.028, *r* = -0.469, correction TP%—KOOSQOL *p* = 0.041, *r* =—0.439).

### Regression analysis

Regression analyses comparing patients without a patellofemoral cartilage lesion to those with one showed that the former were more likely to be younger (*p* = 0.002, Exp(B) 1.139) and have a lower BMI at the time of surgery (*p* = 0.008, Exp(B) 1.26). Patients with a patellofemoral cartilage lesion were more likely to exhibit lower postoperative KOOSpain (*p* = 0.049, Exp (B) 0.001), KOOSsymp (*p* = 0.043, Exp(B) 0.001), KOOS sport (*p* = 0.045, Exp (B) 0.001),KOOS QOL scores (*p* = 0.044, Exp(B) 0.001) (cf. Table 5).

**Table 5:** Regression coefficents, significance levels and odds ratios of all factors analysed

Factor	Regression coefficient	Significance (p)	Odds Ratio {95% CI}
**Age (surgery)**	**0.123**	**0.002**	**1.139 {1.045 – 1.221}**
**Sex**	-0.539	0.438	0.583 {0.141 – 2.039}
**BMI (surgery)**	**0.236**	**0.008**	**1.264 {1.063 – 1.491}**
**Valgus postOP**	-0.306	0.098	0.737 {0.517 – 1.065}
**CDI postOP**	0.495	0.866	1.640{0.005 – 516.086}
**BPI postOP**	-0.805	0.776	0.447 {0.002 – 112.949}
**VAS10y**	0.079	0.721	1.082 {0.701 – 1.672}
**Lysholm10y**	-0.22	0.605	0.978 {0.900—1.064}
**IKDC10y**	-0.009	0.885	0.991 {0.873 – 1.124}
**KOOSpain 10y**	**-7.098**	**0.049**	**0.001 (0.0 – 0.982)**
**KOOSsymp 10y**	**-7.342**	**0.043**	**0.001 {0.0 – 0.809}**
**KOOSadl 10y**	0.732	0.067	2.097 {0.949 – 4.555}
**KOOSsport 10y**	**-7.273**	**0.045**	**0.001 {0.0 – 0.847}**
**KOOSQOL 10y**	**-7.293**	**0.044**	**0.001 {0.0 – 0.825}**
**WOMACpain 10y**	0.624	0.083	1.866 {0.922 – 3.779}
**WOMACstiff 10y**	-0.406	0.146	0.667 {0.385 – 1.153}
**WOMACfunction**	0.969	0.086	2.636 {0.872 – 7.97}
**Tegner 10y**	0.258	0.439	1.295 {0.673 – 2.491}

Significant factors are highlighted in bold

## Discussion

The present study’s main finding is the good to excellent long-term results with an increase of Lysholm score and a decrease of VAS pain levels after HTO for medial compartment osteoarthritis with underlying varus deformity even in the presence of single or kissing cartilage defects in the patellofemoral compartment, with mean patellar indices at or above physiological ranges. Nonetheless, the presence of a cartilage defect in general leads to a lower functional outcome measured by KOOS subscores. In patients with kissing patellofemoral lesions a lower Lysholm score and KOOSQOL subscore than in patients without concomitant patellofemoral cartilage lesion can be expected. Patellar height and extent of valgus correction influence functional outcome in patients with trochlear or kissing lesions. Future studies will be necessary to examine whether correction to mild valgus angles or distally adverted OW-HTO to avoid lowering of patellar height, can further improve functional outcome in these patients. In patients with retropatellar cartilage damage lower functional outcome was associated with the severity of the lesions as well as with female sex.

In summary: In this cohort, patients were corrected to a mild mean valgus of 2.69°. Those without a patellofemoral cartilage lesion were corrected to a slightly larger mean valgus angle (3.16°) than patients with retropatellar (2.2°), trochlear (2.76°) or kissing lesions (2.31°). This valgus correction led to a significant distalisation of the patella, as measured by the CDI and BPI. These findings concur with the published evidence [8, 26–29]. The mean CDI, BPI and ISI indices in all subgroups remained at or above the limit of a patella baja and did not significantly differ among subgroups. In general, cohort patients reported good knee-function levels, low pain levels, little knee stiffness and few limitations in daily living after HTO. While studies addressing geometric changes of the PFJ after HTO have been published, the present study enables HTO’s feasibility to be assessed, for both degenerative PFJ and various cartilage defects (single vs. kissing lesions). Patients in all subgroups significantly profited from HTO and showed significant higher Lysholm scores and lower pain levels (VAS).

Patients with kissing lesions obtained significantly lower Lysholm and KOOSQOL scores than group A patients. The significantly higher patients’ age of group D might have contributed to a lower functional outcome. Presence of a patellofemoral cartilage defect is age-dependent. Nonetheless, all of group D’s WOMAC subscores remained on a good level. Functional outcome of patients with single cartilage lesions of the PFJ did not differ to a significant extent from patients without a cartilage lesion of the PFJ.

A higher Outerbridge Score of the retropatellar lesion was associated with more knee symptoms and pain (KOOSsymp, WOMACstiffness). In the subgroup of trochlear cartilage lesions, a more distalised patella was associated with a higher pain level at final follow-up (VAS). The patella’s distalisation is known to lead to the progression primarily of trochlear cartilage lesions in OW-HTO in short-term follow-up [10, 11, 13] which may explain the rise in pain levels over a longer time period – a fact that might also explain lower Tegner scores in patients with kissing lesions and a lower CDI. A distal tibial tubercle osteotomy (DTO) should thus be considered for these patients as it can prevent a patella infera, lower patellofemoral pressure, and help prevent worsening patellofemoral cartilage defects [11, 12, 15, 17, 30].

In the subgroup of patients with patellofemoral kissing lesions, correction of the weight-bearing axis towards a neutral – slight valgus position was associated with better functional results (Lysholm Score, KOOSpain, KOOSQOL). The influence of valgus correction on the PFJ has been demonstrated. While Otsuki et al. described patellofemoral knee pain following HTO, patients in the present study presented good to excellent functional outcome even though second plane osteotomy was done proximal to the tibial tuberositas [31]. According to Hohloch et al. and Feucht et al., this major finding seems to result from a more gentle correction than in other studies [19, 32].

While the correlation between a larger correction angle and a subsequent increase in retropatellar pressure was proven in a biomechanical cadaver study by Kloos et al., Tanaka et al. supported their finding in a clinical study showing that cartilage injuries tend to worsen with correction angles ± 9 degrees [17, 33].

Yoon et al. reported 39.3% progression of cartilage defects of the trochlea and 23.7% of the patella following HTO. The worst progression was associated with overcorrection exceeding 66.3% on the tibial plateau (highest quartile on the tibial plateau) [13].

Other important evidence derived from our regression analysis showed that the younger the patient and lower their BMI, the less likely they are to suffer patellofemoral lesions. With every year of age and with every point in BMI the chance of a patellofemoral cartilage lesion increases by a factor of 1.139, respectively 1.264. A decrease of KOOS subscore levels increases the chance of the presence of a PFJ lesion. This finding concurs with previous studies demonstrating that OW-HTO can be considered a safe and efficient treatment option for quite young and active patients with medial OA and a varus deformity [34–36].

A limitation of the present study is the lack of a control group, especially a control group of patients after distal tibial tuberosity osteotomy for medial varus OA with patellofemoral cartilage lesions. A large meta-analysis by Kataoka et al. [8] published in 2021 could not prove a causal link of patellofemoral OA and clinical outcomes in patients after OW-HTO. In line with the literature available, cartilage lesions were not monitored by X-ray, MRI or arthroscopy at final follow-up in order to avoid an unnecessary examination and surgery, as well as higher drop-out rates of patients due to change of residence or higher age and reduced mobility. Higher drop-out rates would have increased the risk of bias. Nonetheless, this study delivers the longest follow-up data and thorough analysis of the influence of patellofemoral cartilage lesions on final outcomes after OW-HTO. Furthermore, the present cohort was quite a bit younger than those in comparable studies, and male sex was predominant [8]. The natural aging of patients after a decade might also be associated with lower functional outcome scores and degenerative impairments. Those are factors that might have influenced functional outcome scores.

## Conclusion

Patients with patellofemoral lesions profit from HTO for medial OA with a concomitant varus deformity. Presence of patellofemoral lesions in patients with medial OA and concomitant varus deformity are age-dependent. Especially patients with kissing PF lesions are significantly older and experience significantly lower functional outcome than patients without PF cartilage lesions – nonetheless, even patients with kissing lesions experience a significantly improved functional outcome and reduction of pain level after HTO for medial OA. A neutral to mild valgus correction should be attempted in patients with kissing lesions, and body weight should be controlled to raise functional outcome scores which tend to be lower than in patients with or without single patellofemoral cartilage lesions. In patients presenting larger correction angles and trochlear or kissing lesions of the patellofemoral joint, distal biplanar osteotomy could be considered in order to avoid patellar distalisation.

## Data Availability

The datasets generated and/or analysed during the current study are not publicly available due to the patient data included but are available from the corresponding author on reasonable request if permitted by the competent ethics committee.

## Abbreviations

HTO: High tibial osteotomy
OW-HTO: Open-wedge osteotomy
IKDC Score: International Knee Documentation Committee Score
KOOS Score: The Knee Injury and Osteoarthritis Outcome Score
VAS: Visual Analogue Scale
TKA: Total knee arthroplasty
PFJ: Patellofemoral joint
OA: Osteoarthritis
ACI: Autologous chondrocyte implantation
mLDFA: Mechanical lateral distal femoral angle
mMPTA: Mechanical medial proximal tibial angle
JLCA: Joint line convergence angle
SD: Standard deviation
CDI: Caton-Deschamps-Index
BPI: Blackburne-Peel-Index
ISI: Insall-Salvati-Index
TP: Tibial plateau
WOMAC: Western Ontario and McMaster Universities Osteoarthritis Index
DTO: Distal tubercle osteotomy
